# In Vivo Photoacoustic Imaging of Brain Injury and Rehabilitation by High‐Efficient Near‐Infrared Dye Labeled Mesenchymal Stem Cells with Enhanced Brain Barrier Permeability

**DOI:** 10.1002/advs.201700277

**Published:** 2017-12-05

**Authors:** Weitao Li, Ronghe Chen, Jing Lv, Hongke Wang, Yu Liu, Ya Peng, Zhiyu Qian, Guo Fu, Liming Nie

**Affiliations:** ^1^ Department of Biomedical Engineering College of Automation Engineering Nanjing University of Aeronautics and Astronautics Nanjing 210016 P. R. China; ^2^ State Key Laboratory of Molecular Vaccinology and Molecular Diagnostics and Center for Molecular Imaging and Translational Medicine School of Public Health Xiamen University Xiamen 361102 P. R. China; ^3^ State Key Laboratory of Cellular Stress Biology and Innovation Center for Cell Signaling Network School of Life Sciences Xiamen University Xiamen 361102 P. R. China

**Keywords:** blood–brain barrier, bone mesenchymal stem cells, photoacoustic tomography, Prussian blue, traumatic brain injury

## Abstract

Stem cell migration and interaction with pathology are critical to understand the complexity and status of disease recovery progress. However, the dynamic visualization still remains a great challenge due to imaging technical limitation, cell labeling difficulty, or blood–brain barrier (BBB). Herein, fast photoacoustic tomography (PAT) with optical molecular probes is applied to noninvasively monitor traumatic brain injury (TBI) and its rehabilitation. The vascular distribution and TBI hemorrhage are clearly imaged, longitudinally monitored, and quantified. Bone mesenchymal stem cells (BMSCs) labeled with modified Prussian blue particles (PBPs), excellent near‐infrared dyes and photoacoustic contrasts, are intravenously injected to the mice for improved observation and efficient therapy. BMSCs are demonstrated to be capable of overcoming BBB with enhanced delivery of PBPs to the brain parenchyma. Notably, the versatile BMSCs are observed by PAT to home to the damage region and repair the ruptured vasculature. Moreover, the wound treated by BMSCs exhibits much faster recovery speed than that without treatment. These findings can potentially provide a new noninvasive and high‐resolution approach to image TBI, monitor recovery process, and especially trace BMSCs. This study will stimulate extensive researches on brain diseases and provide promising strategies of dye labeled BMSCs in regenerative medicine.

## 1 Introduction

Traumatic brain injury (TBI) is one of the main causes of mortality in children and young adults.^1^ Several deficits in memory, attention, language, problems solving, and academic skills, and motor functions are among the most common and disabling symptoms of TBI patients.^2^, ^3^ There are numerous methods such as, functional magnetic resonance imaging,^4^ and computed tomography (CT) to diagnose and monitor the craniocerebral injury and its self‐recovery.^5^ However, magnetic resonance imaging (MRI) is only sensitive to deoxyhemoglobin and time‐consuming, preventing it from performing timely therapy monitoring.^6^ While CT is ionizing and less sensitive to the traumatic lesions.^7^ Diffuse optical tomography can reach brain cortex through the scalp and skull beyond the optical scattering limit but lack adequate spatial resolution.^8^

Thus, it is desirable to develop a low‐cost, sensitive, and high‐resolution method to meet these demands. Photoacoustic (PA) tomography (PAT), as a noninvasive imaging modality, possesses not only rich optical contrast but also high ultrasonic resolution.^9^, ^10^ It is a new emerging imaging technique that permits ample contrasts on account of the chromophore absorption.^11^ Recently, PAT has been widely used on both structural and functional imaging of tumor,^12^ microvessels,^13^ brain functions,^14^ vasculature related diseases,^15^ and other applications.^16^, ^17^ Due to these advantages, we employed fast PAT to monitor the damage region of the mouse brain and its recovery with both skull and scalp intact. To confirm the injury, we also acquired CT and MRI of brain. Our findings showed that PAT allowed for monitoring the damaged vasculature with resulting hemorrhage and the rehabilitation process accompanied by blood clot clearance.

Conventional corresponding therapeutic regimen of TBI is to improve intracranial pressure and administrate thrombolytic surgical procedures.^18^ Unlike traditional treatments for brain injury, stem cell therapy is another therapeutic approach to treat TBI because of the self‐renewal and potential to differentiate into various functional cells.^19^, ^20^, ^21^ Moreover, cytokines secreted by bone mesenchymal stem cells (BMSCs), such as neurotropic factors, are beneficial for inducing neovascularization, tissue repair, and neurogenesis in the brain lesion.^22^ Autologous administration of BMSCs was reported to be safe and beneficial to promote neurological recovery in TBI patients.^23^ However, until now the roles of the transplanted stem cells during the whole recovery progress are still indistinct and ambiguous due to the limitations of imaging techniques and probe labeling.

**Scheme 1:**
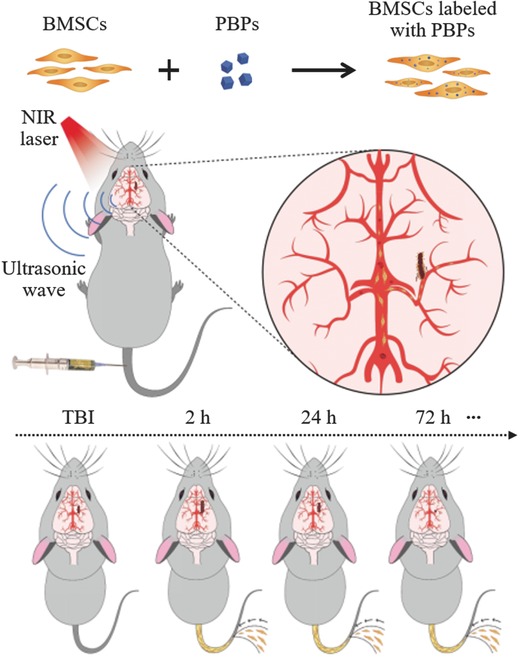
In vivo photoacoustic monitoring of brain injury and rehabilitation by modified Prussian blue particles‐labeled mesenchymal stem cells.

In this study, we established TBI models and used BMSCs to treat cerebral injury with optical imaging and PAT techniques as depicted in **Scheme** **1**. Our experimental results demonstrate that the injected BMSCs have capability of overcoming the blood–brain barrier (BBB) and then migrating to injured region instinctively.^24^ Under normal conditions, the natural recovery process of TBI is typically slow. Surprisingly, mice intravenously administered with BMSCs showed more than a week earlier rehabilitation time and much higher clot removal rate than those without treatment. Furthermore, immunohistochemical staining indicates that numerous regenerated blood vessels were observed at the damage region of the BMSC‐treated group compared with the control group.

Various methods for labeling stem cells include superparamagnetic iron oxide,^25^ radio isotopes,^26^ fluorescent substance,^27^ and quantum dots.^28^ Yet, the BMSCs labeled by optical dyes have rarely been reported nor been explored by PAT. This strategy is similar to previous work on cellular vehicles of near‐infrared (NIR) contrast agents.^29^ Here, citrate‐coated Prussian blue particles (PBPs) were prepared via a simple and improved aqueous synthetic procedure using flash heating^30^ and applied by virtue of its outstanding stability, excellent bio‐compatibility, controllable size distribution, and strong NIR absorption. Furthermore, we investigated in vivo chemotactic movement of BMSCs by using PBPs as dynamic tracers to monitor the healing process of TBI. Our results revealed that a great amount of BMSCs migrated into the injured cerebral tissue. Moreover, BMSCs from the same genotype mice can escape immune system attack and play a transporter role to load PBPs, enabling more tracers to cross the BBB. Compared with unlabeled stem cells, the dye‐labeled BMSCs greatly enhanced the PA signal and image contrast at the lesion.

Collectively, we developed a new approach to noninvasively image the process of TBI, and provided potent strategies on dye‐labeled BMSCs in TBI recovery. PAT results coregistered with fluorescence imaging indicate significant BMSC accumulation in the brain. Compared with in vivo fluorescence imaging, PAT offers more accurate spatial localization with higher resolution, and much deeper penetration depth. Our results demonstrate that PAT aided with high‐efficient NIR dyes is able to trace BMSCs in disease model. This work may also bring a new approach and insight for immunology and regenerative medicine.

## 2 Results

**Figure 1:**
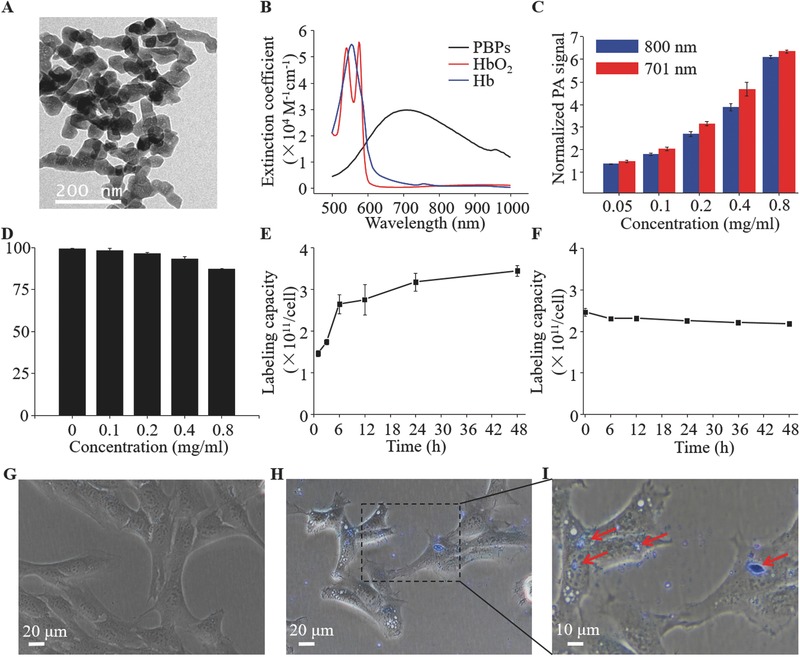
A) TEM image of Prussian blue particles. B) Spectra profiles of PBPs, Hb, and HbO_2_. C) Normalized PA signal amplitude with increased concentrations of the PBPs solution calculated from Figure S2 (Supporting Information). D) Viability of BMSCs treated with different concentrations of PBPs for 24 h. E) Labeling capacity of BMSCs incubated with PBPs for different periods of time. F) Stability of well‐labeled stem cells during 48 h cultivation. The photomicrographs of BMSCs G) before and H,I) after labeling with PBPs. PBPs encapsulated in the BMSCs was indicated by red arrows.

Due to surface coating by citric acid, PBPs exhibit excellent water solubility and stability. The transmission electron microscopy (TEM) showed that the mean diameter of PBPs was about 45 nm (**Figure** **1**A). As shown in Figure 1B, PBPs exhibited a broad absorption band from 600 to 900 nm with an absorption peak of 701 nm. To determine the ability of PBPs as contrast agents, PAT images were acquired from a small animal PA imaging system (Figure S1, Supporting Information). We obtained PA images of PBPs at different concentrations under 701 and 800 nm laser excitation in Figure S2 in the Supporting Information and quantified in Figure 1C. The PA signals increased gradually with the concentration in a linear relationship (*R* ^2^ *_λ_*_= 701 nm_ = 0.92, *R* ^2^ *_λ_*_= 800 nm_ = 0.93).

Methyl thiazolyl tetrazolium assay was conducted in BMSCs to examine the cytotoxicity of PBPs. The results demonstrated no obvious toxicity of PBPs at 0.8 mg mL^−1^ to stem cells after 24 h incubation (Figure 1D). To achieve an appropriate labeling time, BMSCs were cultured in the PBP‐dissolved medium for different time periods. As indicated in Figure 1E, the cell labeling capacity reached a relatively high level (≈2.5 × 10^11^ dye molecules in each cell) when cells were incubated with PBPs for more than 6 h. Besides, the photomicrographs of pure BMSCs and the PBP‐labeled BMSCs were shown in Figure 1G,I. Compared with pure BMSCs, the blue substance internalized by the cell demonstrated that BMSCs were successfully labeled by PBPs. Furthermore, VIS–NIR absorption curve (Figure S3A, Supporting Information) manifested that the absorbance of labeled BMSCs is much greater than the pure BMSCs. PA images (Figure S3B, Supporting Information) were in accordance with the absorption curve, indicating excellent label rate for PBPs to stem cells. Subsequently, labeling stability experiments were performed and the data in Figure 1F showed that less than 10% PBPs leak out of the BMSCs over 48 h, signifying that PBPs labeling was robust within a certain period of time and beneficial for long‐term tracking of stem cells in vitro and in vivo.

PA images of a mouse brain were acquired before and after a single intravenous injection of PBPs solution (0.4 mg mL^−1^, 300 µL). In Figure S4A in the Supporting Information, we can see that the images acquired 10 min after tail vein injection clearly showed the cerebral vessels compared to the preinjection images. This observation became more prominent at 2 h after injection. We circled the middle blood vessel by a box as a region‐of‐interest (ROI) to quantify the PA amplitude value (Figure S4A,B, Supporting Information). Obviously, the improved clarity mainly owes to the added optical absorption by PBPs, suggesting that modified Prussian blue solution could be used as an excellent PAT contrast agent. However, no noticeable signal boost was detected outside the blood vessels.

**Figure 2:**
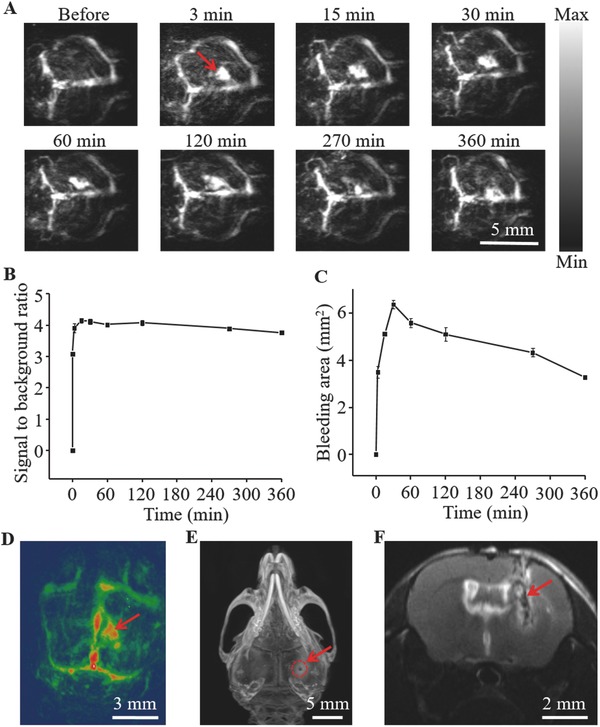
A) PA images of the mouse brain before, 3, 15, 30, 60, 120, 270, and 360 min after needle injury, respectively. The injury area is identified by the red arrow. B) The SBR of the damaged location at the predetermined time points. Error bars represent SEM (*n* = 6). C) The bleeding area change in the damaged region. Error bars represent SEM (*n* = 6). D) 3D PA image of the mouse brain after TBI. E) CT image of the mouse brain after cerebral injury. The wound hole that marked by the red dotted line circle was induced by the steel needle. F) The MRI image (axial plane) of the mouse brain after the brain injury.

A TBI model with vertical needle injury was established on mice to investigate the pathological changes. Before the onset of injury, a PA image of the mice (BALB/c, male, ≈20 g, *n* = 6) brain was illustrated in **Figure** **2**A (“Before” image). Then, PAT of the damaged brain was conducted at predetermined time intervals. As shown in the “3 min” image, a red arrow indicated the injury area. The damage location and the size change of the bleeding area can be clearly visualized at different time points. Furthermore, the coronal, sagittal, and the lambdoidal sutures of the brain marked by the red arrow were obviously mapped in Figure S5A in the Supporting Information. These features were consistent with the photographs of the brain after removing scalp and skull in Figure S5B,C in the Supporting Information, respectively.

In Figure 2B, we quantified the lesion change by circling the bleeding area in an ROI. The signal‐to‐background ratio (SBR) of the damaged region increased dramatically in the first 15 min after TBI due to acute injury to the brain tissue. Afterward, the PA signal value began to decrease gradually because of blood coagulation mechanism. On the other hand, the blood pool began to spread around with PA signal decrease as indicated in Figure 2A. Figure 2C presented the quantitative change of the bleeding area in the damaged region. The bleeding area showed a trend of immediate expansion at the beginning and reached a peak in 30 min. Figure 2D uncovered the 3D PA image of the mouse brain after TBI with a red arrow highlighting the damaged site. The wound hole identified by a red dotted line circle (Figure 2E) in CT image and a circular signal area in MRI image (axial plane) of Figure 2F validated that the brain damage was indeed induced by the inserted needle.

**Figure 3:**
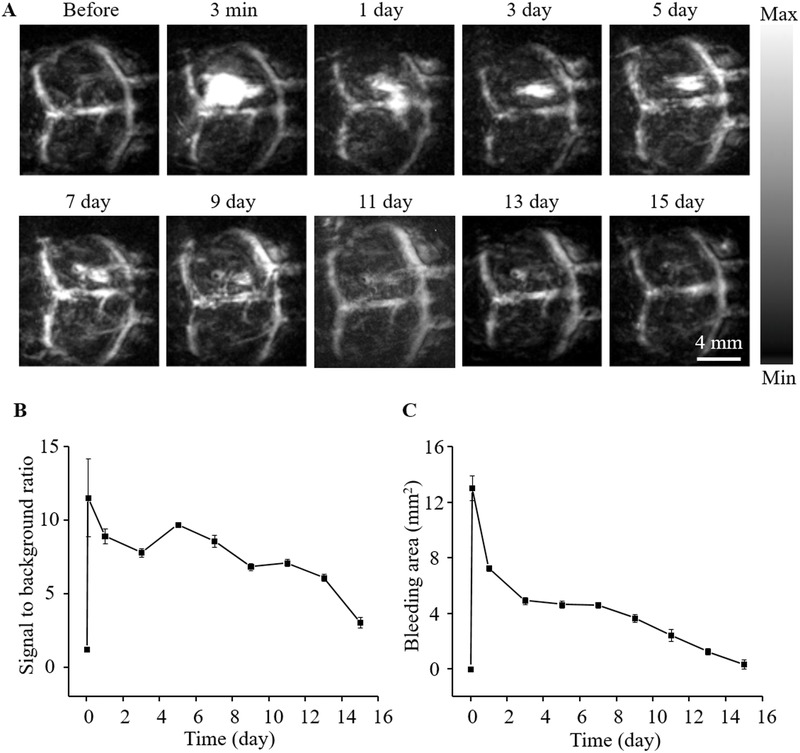
A) PA images of the mouse brain at predetermined time intervals of injury after 3 min, 1, 3, 5, 7, 9, 11, 13, and 15 d, respectively. B) The SBR change of the damaged location at the predetermined time points. Error bars represent SEM (*n* = 6). C) The area change of blood clot in the damaged region at the predetermined time points. Error bars represent SEM (*n* = 6).

To monitor the natural recovery process of mice after craniocerebral injury, PA images were acquired over 15 d with several predetermined time intervals after TBI with horizontal needle injury. As shown in **Figure** **3**A, the damaged region surrounded by the brain hemorrhage could be displayed clearly. The series of PA images revealed a gradual reduction of signal intensity and hematomas from day 0 to 15. On day 13, no noticeable damage or bleeding region could be found in PA image, suggesting self‐clearance after half a month. As can been seen in Figure S5B,C, in the Supporting Information, the pathological status was accordant to a brain biopsy exhibiting small hemorrhagic spot indicated by the white dot circle and strip scar surrounded by the white dash box. Figure 3B showed the SBR change of the damage region at different time points calculated from Figure 3A. At the same time, the area of the damage zone decreased gradually in Figure 3C. These PA images indicated that the recovery process of the abnormality could be fully monitored by using fast PAT.

We also sought to monitor therapeutic efficacy of BMSCs on mice brain after cerebral injury. Female BALB/c mice weighing 20–22 g were induced TBI and randomly divided into control, BMSC‐treated, and PBP‐labeled BMSC‐treated groups. The TBI model was completed based on the horizontal injury as mentioned earlier. The mice in both treatment groups were administrated with three consecutive intravenous injections of BMSCs (1 × 10^6^, 200 µL) labeled or unlabeled with PBPs at 2, 24 and 72 h after induction of brain injury. The control group mice were injected with the equivalent volumes of phosphate‐buffered saline (PBS) at the same time points. Then, PA images were collected to study the treatment process by BMSCs after TBI in mice.

**Figure 4:**
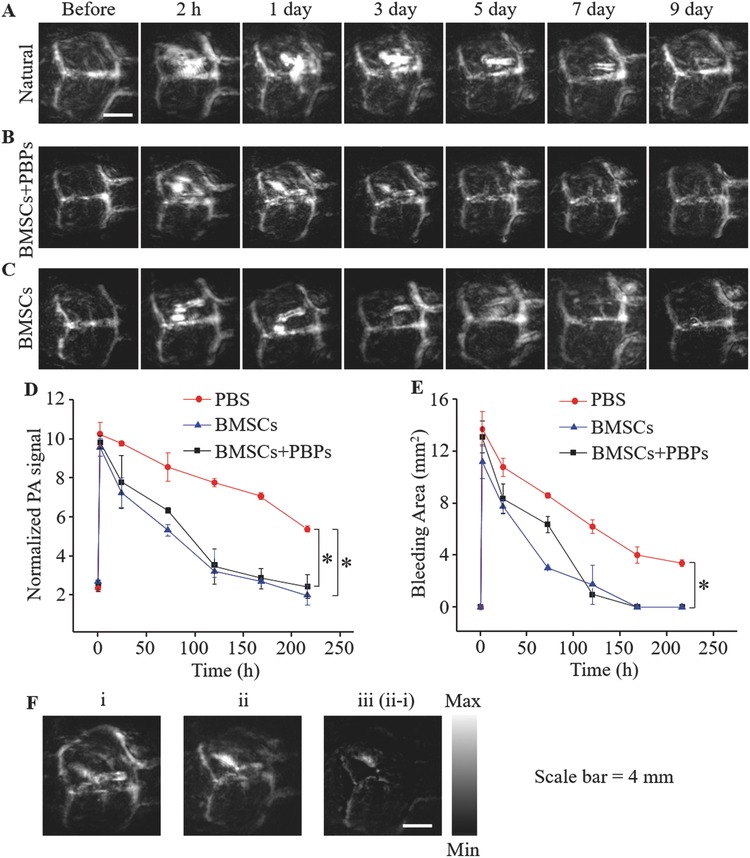
A) Mouse brain PA images of control group, B) PBP‐labeled BMSC‐treated, and C) BMSC‐treated groups at the predetermined time intervals of the recovery process. D) Normalized PA signal intensities of the damaged location at the predetermined time points. Error bars represent SEM (*n* = 6 per group). *P < 0.05 compared to PBS group. E) The blood clot area in the damaged region. Error bars represent SEM (*n* = 6 per group). *P < 0.05 compared to PBS group. F) PA images of the mouse brain (i) before and (ii) after a single injection of BMSCs (1 × 10^6^, 200 µL) labeled with PBPs (0.1 mg mL^−1^). The image (iii) was obtained by subtracting the before injection image from the after injection image (iii = ii − i).

During the whole process, we implemented a timely monitoring to the TBI mice for a series of PA images. As shown in **Figure** **4**A, mice in the control group took ≈2 weeks in the natural repair process. However, mice in the treatment group (Figure 4B,C) recovered in 5 d and the blood clots were cleared thoroughly. In Figure 4D, we quantified the PA signals of the damaged region in Figure 4E showing the area change of the blood clot. From Figure 4D,E, we found that PA signals and blood clot areas in the damaged region reduced gradually and finally disappeared as time lapsed. The slopes of normalized PA signal and the blood area curves of both stem cells treated groups were sharper than that of the control group. Especially, PBP‐labeled BMSC group recovered slightly slower than only BMSC group.

This phenomenon is probably attributable to the signal contribution of exogenous dye. The experimental results disclosed that the BMSCs indeed promoted the recovery of the broken vessels and the elimination of blood clots. PA images (λ = 800 nm) of the mice brain before and after injection of PBP‐labeled BMSCs were also obtained to validate the cell accumulation to the injury site. The PA signal of the damaged region 10 min after the injection (Figure 4Fii) was higher than that before the injection (Figure 4Fi) with subtracted image shown in Figure 4Fiii, implying that the labeled stem cells homed, or circulated to the injury site.

**Figure 5:**
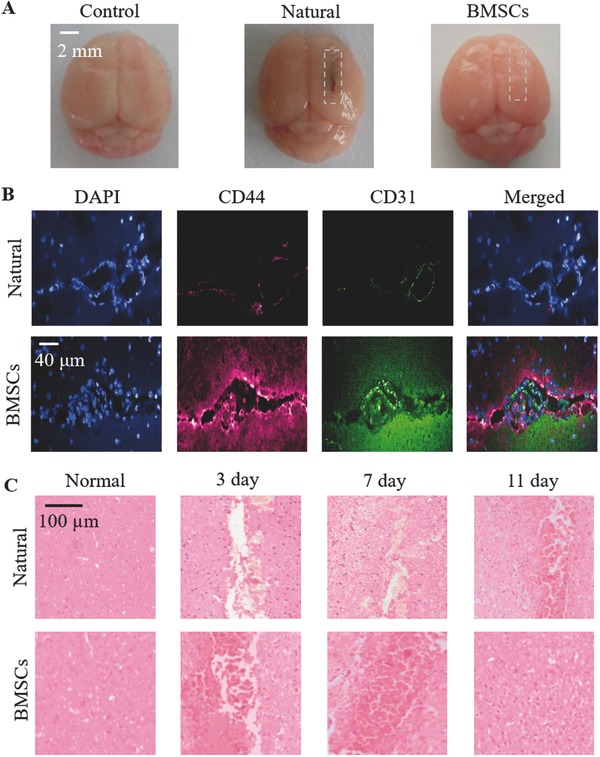
A) Photographs of the whole brain tissues before and 7 d after TBI. The dashed white box indicated the injured area. B) DAPI and immunohistochemical staining of brain sections for CD31 and CD44 in injured region after BMSC therapy of TBI. Blue, aubergine, and green fluorescence correspond to DAPI, CD44, and CD31, respectively. C) H&E staining of brain tissue slices during the recovery progress of TBI with or without intravenous administration of BMSCs.

Brain tissues were excised to further evaluate the rehabilitation progress of TBI. The sections were stained with 4′,6‐Diamidino‐2‐Phenylindole (DAPI), anti‐CD31 and anti‐CD44 antibodies, and hematoxylin and eosin (H&E) at 7 d after TBI. The photographs of dissected brain tissues are shown in **Figure** **5**A. Compared with the natural recovery mice, the wound of BMSC‐treated mice nearly healed as the normal mice. In Figure 5B, the fluorescence intensity around the cerebral vessels was much higher in the BMSC‐treated group than in the natural group, implying the obvious neovascular growth, and stem‐cell accumulation in the damaged region of BMSC‐treated mice. Besides, H&E staining of brain tissue slices also revealed enhanced gliosis and increased cellular density in BMSC therapy in comparison with obvious broken fissures in natural rehabilitation (Figure 5C). Therefore, these results illustrate that BMSCs flock to the lesion and significantly contribute to wound angiogenesis.

**Figure 6:**
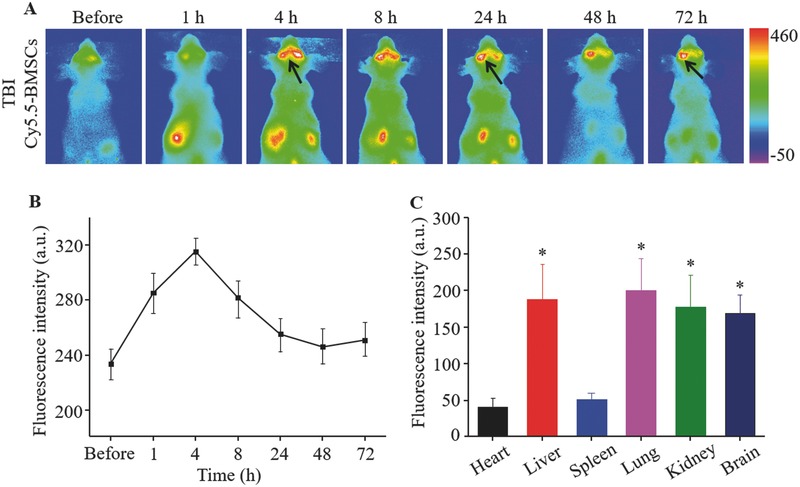
A) Fluorescence imaging of the mice injected with Cy5.5‐labeled BMSCs. B) Quantitative fluorescence intensity of the damage region in TBI mice after intravenous injection of Cy5.5‐labeled BMSCs. Error bars represent SEM (*n* = 6). C) Quantitative fluorescence intensity of major organs including heart, liver, spleen, lung, kidney, and brain 72 h postinjection of Cy5.5‐labeled BMSCs. Error bars represent SEM (*n* = 6). *P < 0.05 compared to heart or spleen.

In a further study, we used in vivo fluorescence imaging to trace the fate of transplanted stem cells. While inoculating with PBPs, BMSCs were simultaneously labeled with Cy5.5, a near‐infrared fluorescence‐emitting dye. The photomicrographs of BMSCs before and after marking Cy5.5 were presented in Figure S6A (Supporting Information). The kermesinus signal accumulated in stem cells demonstrated that BMSCs were successfully labeled with Cy5.5. In Figure S6B,C (Supporting Information), the fluorescence signal produced by Cy5.5 was linearly dependent on the concentration. As shown in **Figure** **6**A and B, the Cy5.5‐labeled BMSCs displayed strong migration tendency to the damage region at 4 h after injection. This trend reduced gradually and remained present at 72 h, indicated by black arrows. The fluorescence imaging data were consistent with the PA results.

We further investigated the biodistribution of Cy5.5‐labeled BMSCs in TBI mice organs after injection. All mice were sacrificed and main organs were harvested for the ex vivo fluorescence imaging. From Figure 6C, a lot transplanted stem cells were entrapped by organs such as lung and liver, which was also previously observed during intravenous administration.^19^, ^31^ However, a great number of BMSCs still migrated to the brain for therapeutics.

## 3 Discussion

In this study, we used PAT to noninvasively monitor the damage region of the mouse brain and its rehabilitation process after TBI. Our results showed that PAT clearly imaged the vascular distribution and fully monitored the rehabilitation of the craniocerebral injury. PAT was capable of providing high‐resolution and high‐sensitivity pathological information. Total hemoglobin concentration and the change of bleeding area in acute brain injury were obtained, which reflected the bleeding and physiological status in the region of the lesion.

Stem cells can differentiate into various functional cells and have therapeutic capabilities in many diseases.^32^, ^33^, ^34^ Thus, a suitable tracer marking the MSCs is important to demonstrate the implantation and migration of transplanted stem cells.^35^ We applied biocompatible and high‐absorbing PBPs to label BMSCs and made them as tracers to monitor the healing process of TBI. Our experiments have illustrated that the labeled stem cell can home to the diseased site and the enhanced PA signals are contributed by NIR dyes. Obviously, the blood clot clearance rate of the treatment group intravenously administrated of MSCs was much faster than the control group after TBI. The injured brain treated with BMSCs only required a recovery period of 5 d after TBI, which is less than half of the control group's time. From the histological examination, enhanced gliosis and angiogenesis were found in the damage region of BMSC‐treated mice. The results suggested that stem cell therapy significantly contributed to the rehabilitation of TBI model. While the PA images and pathologic analyses are seemingly convincing, neurobehavioral examinations before and after BMSC treatment, such as general health, motor functions, sensory abilities, memory and learning, are under way.

In vivo fluorescence imaging was also applied to trace the BMSC fate by labeling Cy5.5 to further verify the cell homing. The fluorescence imaging data indicated that a part of intravenously administered Cy5.5‐labeled BMSCs indeed homed into the TBI site and this migration tendency remained as long as 72 h after injection. Besides, the biodistribution analysis of Cy5.5‐labeled BMSCs revealed that a large proportion of the transplanted stem cells were entrapped by the lung and other organs after intravenous injection. Similar results were observed but most of the administration stem cells would not bring apparent harm.^36^, ^37^

## 4 Conclusion

In summary, we demonstrate that PAT can be not only applied to noninvasively monitor the mouse brain injury and its rehabilitation but also used for stem cells tracing in the pathological area. The PBPs, excellent NIR dyes and PA contrasts, have ability to label BMSCs and track the fate of stem cells. Stem cells as transporters loaded with PBPs allow more tracers to home to the lesion site, which largely improve brain injury observation. This Trojan method can escape immune system attack and deliver more agents to the injured site, which will stimulate more similar work. Our work can potentially provide a new strategy of NIR dye labeled stem cells in brain disease study and open up a new application of PAT in regenerative medicine.

## 5 Experimental Section

*Reagents*: Unless otherwise noted, all solvents and compounds were purchased from Aldrich Chemical Co. (St. Louis, MO). Cy5.5‐NHS ester was purchased from GE Healthcare Life Science (Piscataway, NJ). Primary antibody anti‐CD31 and anti‐CD44 were purchased from Abcam (Cambridge, MA). Secondary antibody FITC‐labeled goat antirabbit IgG (H + L) and Cy3‐labeled goat antirat IgG (H + L) were purchased from Beyotime (Shanghai, China).

*Small Animal PA Imaging System*: Hemispherical PA imaging system (Nexus 128 scanner, Endra Inc., Ann Arbor, MI) was applied to monitor the mice brain. The schematic of this system is shown in Supplementary Figure 1. The laser wavelength is tunable from 680 to 950 nm for PA signal excitation. A planoconcave lens was used for laser beam expansion. The incident energy density on the mouse brain surface was about 0.86 mJ cm^−2^, which is well below the American National Standard Institute (ANSI) safety threshold, 20 mJ cm^−2^. The mouse was held by a custom‐built plastic tray. A water circulation system was used to maintain the temperature of the water at 37 °C to avoid hypothermia in mice. Ultrasound detection was achieved through a hemispherical ultrasonic device that consists of 128 ultrasonic transducers. Then, the PA signals were transferred to a computer for data reconstruction. A complete circular scan usually takes ≈1.7 min. The spatial resolution of this imaging system is about 200 µm.^38^

*Animal Preparation*: All in vivo animal experimental procedures were approved by the Institutional Animal Care and Use Committee of Xiamen University. BALB/c mice weighing about 20–22 g were used in this study. Before the experiment, the fur of the mice head was shaven and chemically depilated. During PAT data acquisition, the mouse was placed in the animal tray and some ultrasonic coupling gel was smeared on the surface of the mice head for better transmission of the ultrasonic signal to the ultrasonic sensor. The mouse needs to be placed at the bottom of the tray and kept still by using a breathing isoflurane anesthesia system. The bottom of the tray immersed in ultrapure water and the temperature of the water was kept at 37 °C. The location of the mouse head is placed at the same position to guarantee consistent measurements.

*Mice Brain Injury Modeling*: A steel needle with a length of 38 mm and maximal diameter of 0.5 mm was used to establish two injury models. In the first model, the steel needle was inserted vertically to a depth of 3 mm underneath the right scalp of the mouse for 3–5 s to induce a traumatic lesion. In the second model, the steel needle was horizontally inserted to the brain. The insertion length of the needle was about 5 mm. Moreover, the mouse was kept alive during the experiment and the steel needle was pulled out after the damage.

*Preparation of PBPs*: PBPs were synthesized according to previous literatures with some modifications.^30^, ^39^ Briefly, 0.5 mmol citric acid monohydrate (105 mg) was added into 20 mL FeCl_3_ aqueous solution (1.0 × 10^−3^ m) under stirring at 60 °C. Then, 20 mL K_4_[Fe(CN)_6_] aqueous solution (1.0 × 10^−3^ m) containing 0.5 mmol citric acid monohydrate was added to the above solution under stirring at 60 °C for 30 min. During the mixing process, a clear bright blue dispersion formed gradually and cooled to room temperature under stirring for another 30 min. After that, 4 mL product mixture was added to a 100 K filter (Millipore, MA) under centrifugation at 11 000 rpm for ≈15 min with three times repetition to remove excess FeCl_3_ solution and other small molecules. Then, the residue that contains only PBPs precipitation was resuspended in 4 mL PBS. Before utilization, the solution was further filtered through a 0.22 µm filter to avoid particle aggregation.

*Cultivation and Labeling of BMSCs*: The primary BMSCs were acquired from the same genotype mice (Cyagen Biosciences Inc.) and were seeded in a culture dish with 10 mL MSC growth Dulbecco's modified Eagle's medium (MSCGDMEM, OriCellBalb/c, Cyagen Biosciences Inc.). Culture media was replaced at 2 d intervals. When the BMSCs reached a density of 1 × 10^6^ cells/dish, the medium was replaced with 4 mL fresh MSCGDMEM. Then, equal volumes of 0.2 mg mL^−1^ PBPs were added to the above cell‐culture medium. To label BMSCs with PBPs, BMSCs were incubated with the mixture for different periods of time (1, 3, 6, 12, 24, and 48 h) at 37 °C and in a 5% CO_2_ environment. After incubation, the culture supernatant was collected and the residual PBPs without internalization were obtained after three times PBS (pH = 7.4) washing. Then, PBPs absorption of the above retained liquid was measured by a spectrometer at 705 nm. PBPs content was calculated from the standard curve constructed with increasing concentrations of PBPs in MSCGDMEM solution. Thus the cell labeling capacity was indirectly computed via subtracting the unabsorbed PBPs from the initial PBP level. All the measurements were performed at least in three independent samples. Meanwhile, the live imaging of PBP‐labeled BMSCs was conducted on an optical microscope to further confirm intracellular internalization. Finally, the well‐labeled BMSCs were resuspended in PBS for biological usage.

*Labeling Stability*: To investigate stability of PBP labeling, the well‐labeled stem cells were cultured in serum containing medium at 37 °C for 48 h. At predetermined times (0, 6, 12, 24, 36, and 48 h), the supernatant was collected to determine PBPs content using VIS spectrophotometry based on the standard curve. Similarly, the labeling capacity of BMSCs was calculated in the indirect way as previously described and the capacity change was observed for stability evaluation.

*In Vivo Fluorescence Imaging*: Fluorescence imaging was carried out in Carestream FX PRO (Carestream Health Inc., Toronto, Canada). BALB/c nude mice were anesthetized by breathing isoflurane and immobilized. After that, the mice were irradiated by using 630 nm laser. The white‐light images and luminescence images (exposure time, 20 s) were captured individually. For PA imaging in vivo, BALB/c mice induced with TBI model were injected with unlabeled and labeled BMSCs (200 µL, 1 × 10^6^), followed by anesthetization and PA imaging at predetermined time points.

*CT Imaging*: The CT imaging was performed by using a CT preclinical scanner (Inveon PET/CT, Siemens) with the following acquiring parameters: energy peak of 140.5 keV, window width of 20%, matrix of 256 × 256, medium zoom, and frame of 30 s.

*In Vivo MRI Imaging*: In vivo T2‐weighted images of TBI mouse was determined using a 7.0 T MR scanner (Biospec USR70/20, Bruker) by using the multislice multiecho sequence with the following parameters: repetition time/echo time: 4000/9.5 ms; echo image: 10; slice thickness: 0.5 mm; field of view: 2 × 2 cm; matrix: 256 × 256.

*Histological Analysis*: At the first, mice were euthanized at the predetermined time points. After that, they were perfused intracardially with 60 mL of 4% paraformaldehyde. The brains were removed and stored in 4% paraformaldehyde. The right brain damage areas were dissected and embedded in paraffin. A series of adjacent 5 µm horizontal sections of the brain injury region were cut and stained with H&E. On average, five slices per brain were prepared. The photograph was obtained at 200× magnification using an optical microscope equipped with a digital camera.

*Immunohistochemical Staining*: At predetermined time points, mice were euthanized and were perfused with 40 mL physiological saline. After that, brains were removed and immersed in 4% paraformaldehyde solution. The fixed brains were placed in 30% sucrose solution overnight before being embedded in Optimum Cutting Temperature (OCT) medium for snap freezing histological analysis. Afterward, 10 µm thick brain tissue slices were obtained by cryotome sectioning. The slides were taken out and dried for 15 min at room temperature. The slides were washed three times using 1 × PBS and blocked using 10% sheep serum (in PBS with 5% BSA) for 1 h at room temperature. Primary antibody CD31 (1:200) and CD44 (1:200) in PBS with 5% BSA were added to the slides and incubated overnight at 4 °C. Then we washed the slides three times using 1 × PBS. After that, we added secondary antibody FITC‐labeled goat antirabbit IgG (H + L) and Cy3‐labeled goat antirat IgG (H + L) (1:500 in PBS with 5% BSA) in dark room and incubated slides in dark room 1 h and then washed three times using 1 × PBS. We mounted the slides using mounting medium and stored them in 4 °C dark box. Tissue sections were photographed with the Opera Phenix high‐content screening System using a 40 × oil immersion objective.

*Statistics*: Quantitative results were expressed as mean ± standard error of mean (SEM). Statistical analyses were performed using SPSS 18.0 software. Nonparametric data were analyzed with the Kruskal–Wallis test and the Nemenyi multiple comparison test. Parametric data were compared by ANOVA and the Tukey post hoc test. A *P* < 0.05 was accepted as statistically significant.

## Conflict of Interest

The authors declare no conflict of interest.

## Supporting information

Supplementary
