# Supplementary material for: In Vivo Photoacoustic Imaging of Brain Injury and Rehabilitation by High‐Efficient Near‐Infrared Dye Labeled Mesenchymal Stem Cells with Enhanced Brain Barrier Permeability
Source: Adv Sci (Weinh). 2017 Dec 5;5(2):1700277. doi: 10.1002/advs.201700277 (PMC5827566; doi:10.1002/advs.201700277)
Supplement: Supplementary file 1 — Supplementary [file ADVS-5-1700277-s001.pdf]

Copyright WILEY-VCH Verlag GmbH & Co. KGaA, 69469 Weinheim, Germany, 2017.

Supporting Information

for *Adv. Sci.,* DOI: 10.1002/advs.201700277

In Vivo Photoacoustic Imaging of Brain Injury and Rehabilitation by High-Efficient Near-Infrared Dye Labeled Mesenchymal Stem Cells with Enhanced Brain Barrier Permeability

*Weitao Li, Ronghe Chen, Jing Lv, Hongke Wang, Yu Liu, Ya Peng, Zhiyu Qian, Guo Fu, and Liming Nie**

Copyright WILEY-VCH Verlag GmbH & Co. KGaA, 69469 Weinheim, Germany, 2016.

Supporting Information

In Vivo **Photoacoustic Imaging of Brain Injury and Rehabilitation by High-Efficient NIR Dye Labeled Mesenchymal Stem Cells with Enhanced Brain Barrier Permeability**

*Weitao Li, Ronghe Chen, Jing Lv, Hongke Wang, Yu Liu, Ya Peng, Zhiyu Qian, Guo Fu, and Liming Nie**

Dr. W. Li, H. Wang, Prof. Z. Qian
Department of Biomedical Engineering
College of Automation Engineering
Nanjing University of Aeronautics and Astronautics
Nanjing 210016, P. R. China

Dr. R. Chen, J. Lv, H. Wang, Y. Liu, Y. Peng, Prof. L. Nie
State Key Laboratory of Molecular Vaccinology and Molecular Diagnostics & Center for Molecular Imaging and Translational Medicine
School of Public Health
Xiamen University
Xiamen 361102, P. R. China
E-mail: nielm@xmu.edu.cn

Prof. G. Fu
State Key Laboratory of Cellular Stress Biology and Innovation Center for Cell Signaling Network
School of Life Sciences
Xiamen University
Xiamen 361102, P. R. China

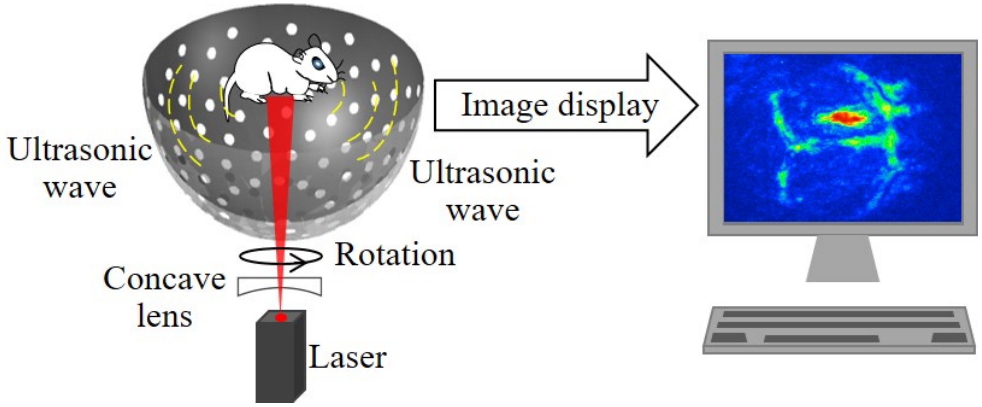

Figure S1 Schematic illustration of small animal photoacoustic imaging system.

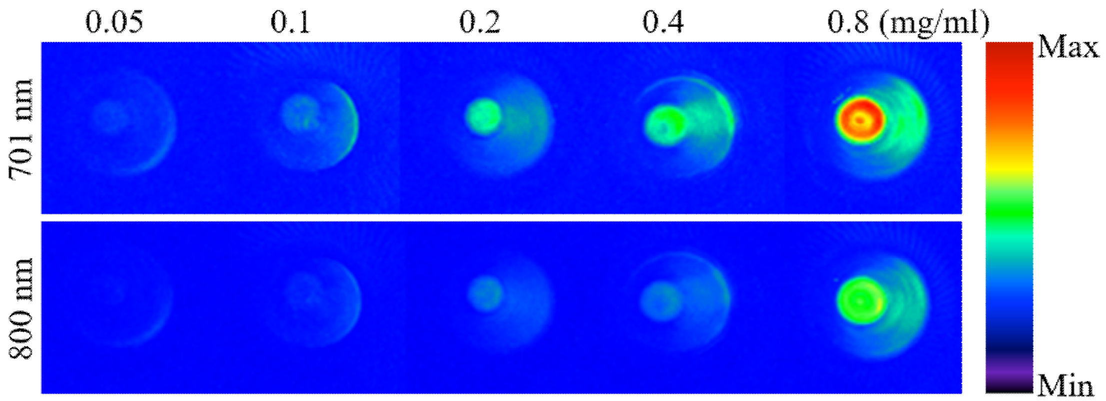

Figure S2 PA images of PBPs solution at 701 nm and 800 nm at different concentrations.

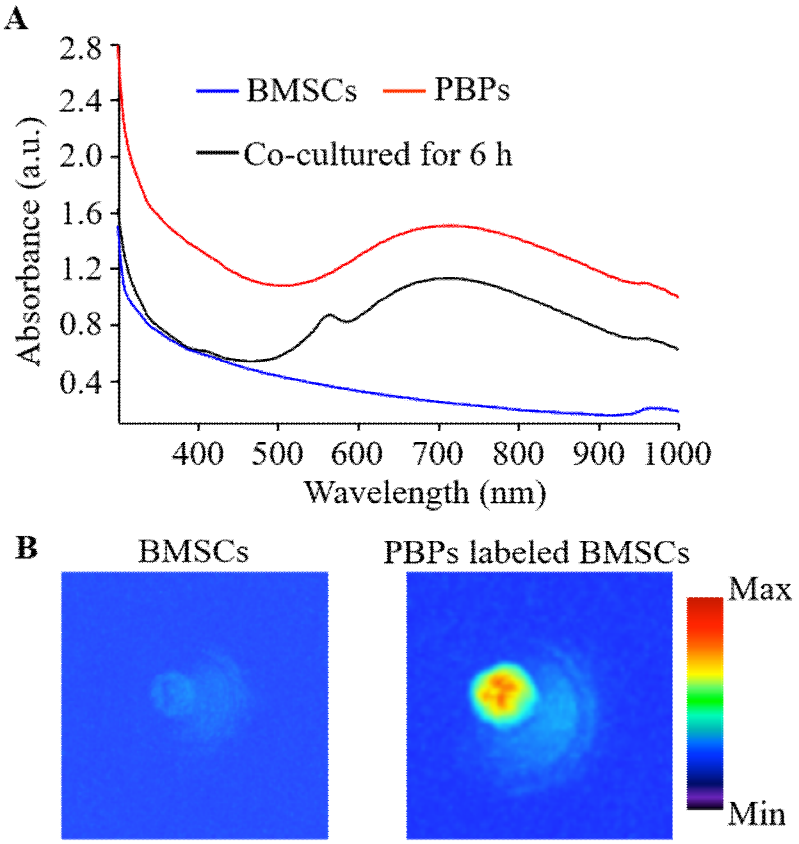

Figure S3 (**A**) Absorption spectrum of BMSCs, PBPs and BMSCs labeled with PBPs. (**B**) PA imaging of BMSCs and PBPs labeled BMSCs.

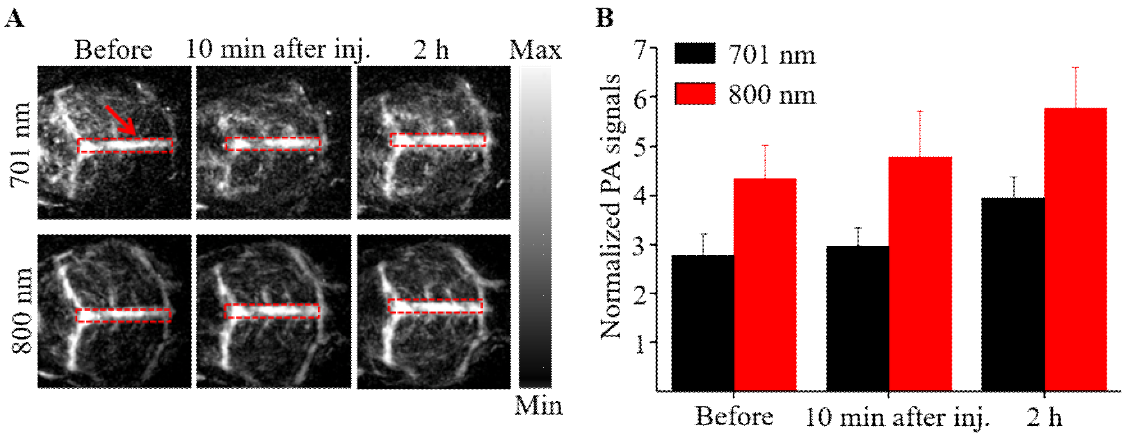

Figure S4 (**A**) PA images of the mouse brain before and after an injection of PBPs solution (0.4 mg/ml, 300 μl). (**B**) Plot of the normalized photoacoustic signal amplitude at different time points: before the injection, 10 minutes after injection and 2 hours after injection.

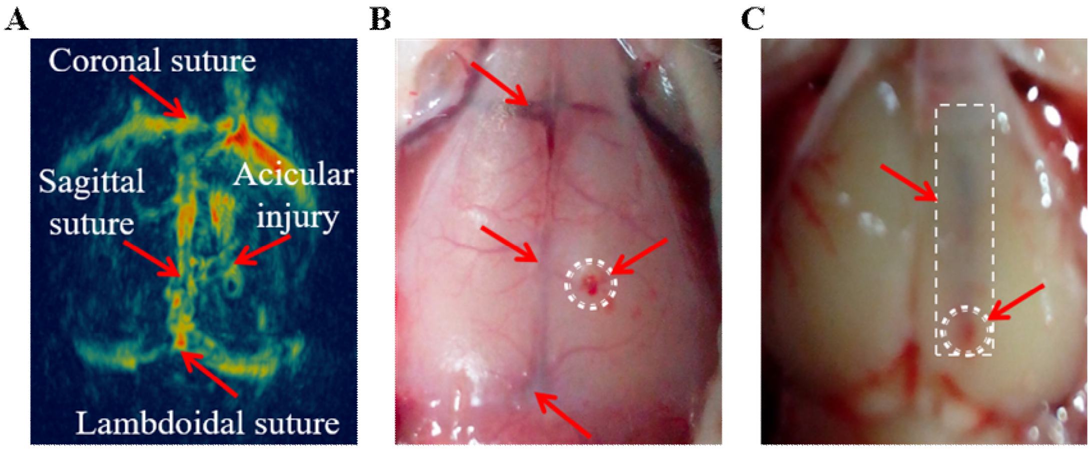

Figure S5 PA image and photograph of the mouse after brain injury. (**A**) A view of 3D PA image of the mouse after brain injury. The mouse was executed and (**B**) scalp of the brain was removed immediately and (**C**) the skull was taken out afterwards.

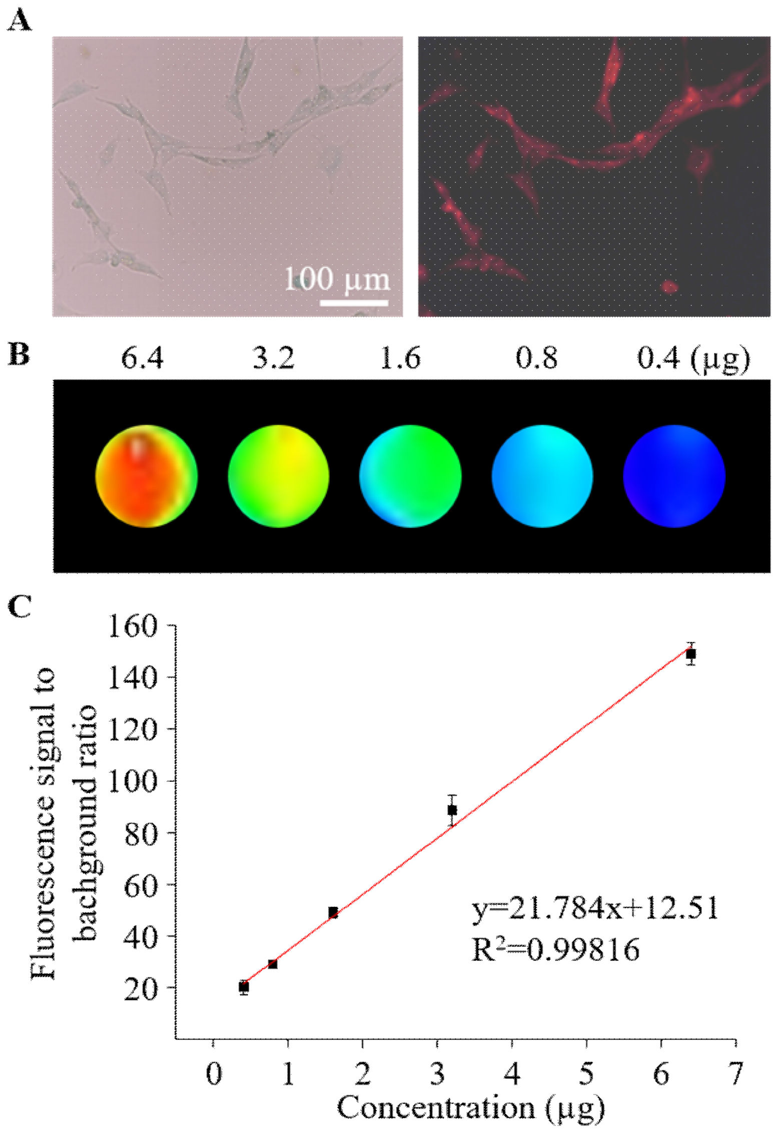

Figure S6 (**A**) Photomicrograph of BMSCs before and after labeling with Cy5.5. (**B**) Fluorescence images of Cy5.5 at different concentrations. (**C**) Plot of the fluorescence signal to background ratio with increasing mass of Cy5.5.
